# EQClinic: a platform for learning communication skills in clinical consultations

**DOI:** 10.3402/meo.v21.31801

**Published:** 2016-07-29

**Authors:** Chunfeng Liu, Karen M. Scott, Renee L. Lim, Silas Taylor, Rafael A. Calvo

**Affiliations:** 1 School of Electrical and Information Engineering, The University of Sydney, Sydney, NSW, Australia; 2 Discipline of Paediatrics and Child Health, Sydney Medical School, The University of Sydney, Sydney, NSW, Australia; 3 Sydney Medical School, The University of Sydney, Sydney, NSW, Australia; 4 Office of Medical Education, UNSW Medicine, UNSW Australia, Sydney, NSW, Australia

**Keywords:** communication skills training, non-verbal communication, tele-consultation, medical education, non-verbal behaviour recognition, automated feedback

## Abstract

**Background:**

Doctors’ verbal and non-verbal communication skills have an impact on patients’ health outcomes, so it is important for medical students to develop these skills. Traditional, non-verbal communication skills training can involve a tutor manually annotating a student's non-verbal behaviour during patient–doctor consultations, but this is very time-consuming. Tele-conference systems have been used in verbal communication skills training.

**Methods:**

We describe EQClinic, a system that enables verbal and non-verbal communication skills training during tele-consultations with simulated patients (SPs), with evaluation exercises promoting reflection. Students and SPs can have tele-consultations through the tele-consultation component. In this component, SPs can provide feedback to students through a thumbs-up/ thumbs-down tool and a comments box. EQClinic automatically analyses communication features in the recorded consultations, such as facial expressions, and provides graphical representations. Our 2015 pilot study investigated whether EQClinic helped students be aware of their non-verbal behaviour and improve their communication skills, and evaluated the usability of the platform. Students received automated feedback, and SP and tutor evaluations, and then completed self-assessment and reflection questionnaires.

**Results:**

Eight medical students and three SPs conducted 13 tele-consultations using EQClinic. More students paid attention to their non-verbal communication and students who were engaged in two consultations felt more confident in their second consultation. Students rated the system positively, felt comfortable using it (5.9/7), and reported that the structure (5.4/7) and information (5.8/7) were clear. This pilot provides evidence that EQClinic helps, and positively influences, medical students practise their communication skills with SPs using a tele-conference platform.

**Discussion:**

It is not easy to improve non-verbal communication skills in a short time period. Further evaluation of EQClinic with larger numbers will ascertain learning gains and application in health professional training. Developing a standard model for the assessment of non-verbal behaviour in tele-consultations and providing students with more valuable evaluation and suggestions are part of our future work.

There is a broad agreement about the importance of clinical communication skills training in medical education (1), given the evidence that clinical communication skills influence patient health outcomes (2). According to social cognitive theory, students acquire competence through practice and feedback (3); therefore, to develop clinical communication skills, medical students need to practise with real or simulated patients (SPs) and receive feedback from patients and tutors. Some medical schools provide students with video recordings of their interactions with patients, which positively influence the development of their clinical communication skills (4). However, the cost and time of organising a large number of practice sessions and setting up the recording environment are a challenge. Tele-conference has been proposed as a solution for dealing with this challenge (5). The WebEncounter tele-conference platform, developed to enable medical interns to communicate with SPs, showed that practising on WebEncounter enhanced the communication skills and adeptness of interns in giving bad news (6).

Like WebEncounter, most clinical communication skills training systems focus on the verbal content of consultations. Non-verbal communication, which accounts for 65–95% of essential communication between individuals (7, 8), is often not given sufficient attention. Providing students with manually annotated feedback about their non-verbal behaviour can benefit the learning of non-verbal communication skills (9), but the time-consuming nature of these methods prevents them from being widely incorporated into the teaching curriculum. Training platforms have been developed to provide medical professionals with real-time feedback about their non-verbal behaviour in face-to-face consultations, although the feedback was manually annotated by observers (10). In research into social skills training, a system has been developed to sense non-verbal behaviour and provide feedback for reflection (11). However, there has been little research into the use of such novel technologies for communication skills training during medical education.

Recent research has suggested that the use of telehealth to deliver education and training has not received enough attention (12, 13). Our broad research programme proposes an innovative clinical communication skills training platform, EQClinic (14), which can automatically identify the non-verbal behaviour of medical students in a telehealth consultation and provide feedback for reflection (15). Given the five determinants of successful telehealth implementations are technology, acceptance, financing, organisation, and policy and legislation (16), in this first development phase, we need to evaluate the technical stability and user acceptance of the system's initial design (17). Thus, the purpose of this pilot study was to conduct research that demonstrated EQClinic's system stability and acceptance by students when used as a teaching tool. In addition, we explored whether EQClinic would help medical students identify their non-verbal behaviour and improve their communication skills.

## Methods

### EQClinic platform

EQClinic was developed by the Positive Computing Lab at the University of Sydney, Australia, in collaboration with medical schools at the University of Sydney and University of New South Wales, Australia. EQClinic aims to: 1) provide medical students with opportunities to communicate with SPs via tele-consultation and an easy means of organising the consultations; 2) provide video recordings of the consultations, with different types of feedback, including assessments and comments from SPs and tutors; and 3) automatically identify students’ non-verbal behaviour, such as pitch, facial expressions, and body movements, using audio processing and computer vision techniques, and provide graphical representations of these for students after each tele-consultation. EQClinic includes two surveys to facilitate student reflection, which is crucial for learning according to the Kolb learning cycle (18).

EQClinic consists of three main components: a personal calendar, a tele-consultation component, and a feedback generator. Students and SPs use the automated personal calendar system to book tele-consultations. SPs can offer their available time slots on the calendar to allow students to make a booking. EQClinic uses different colours to label the changing status of the appointments, so that students can easily request and check the status of any available time slot. Email and SMS notifications are sent from the system, when users request or confirm appointments.

**Fig. 1:**
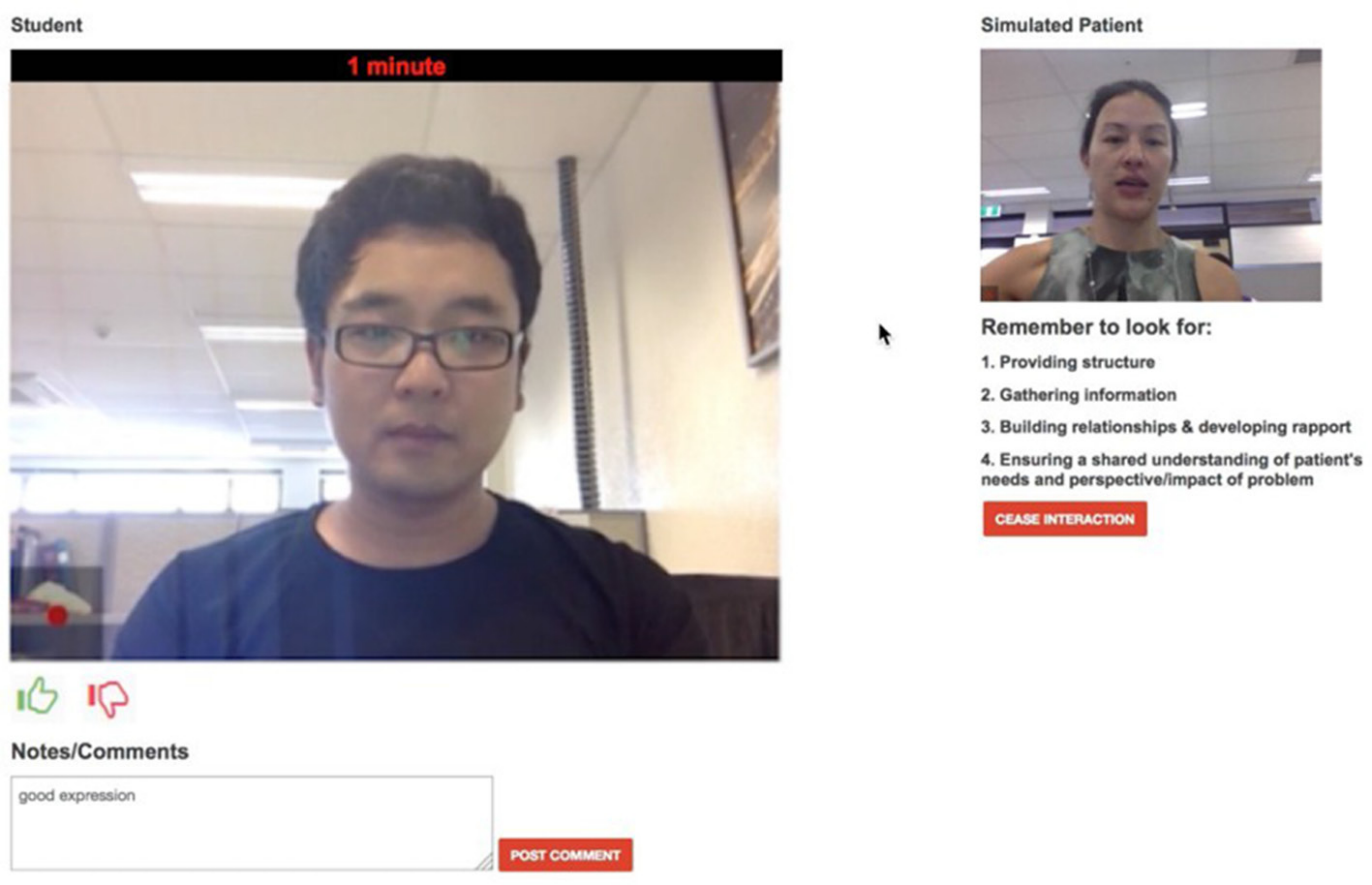
SP's tele-conference page.

**Fig. 2:**
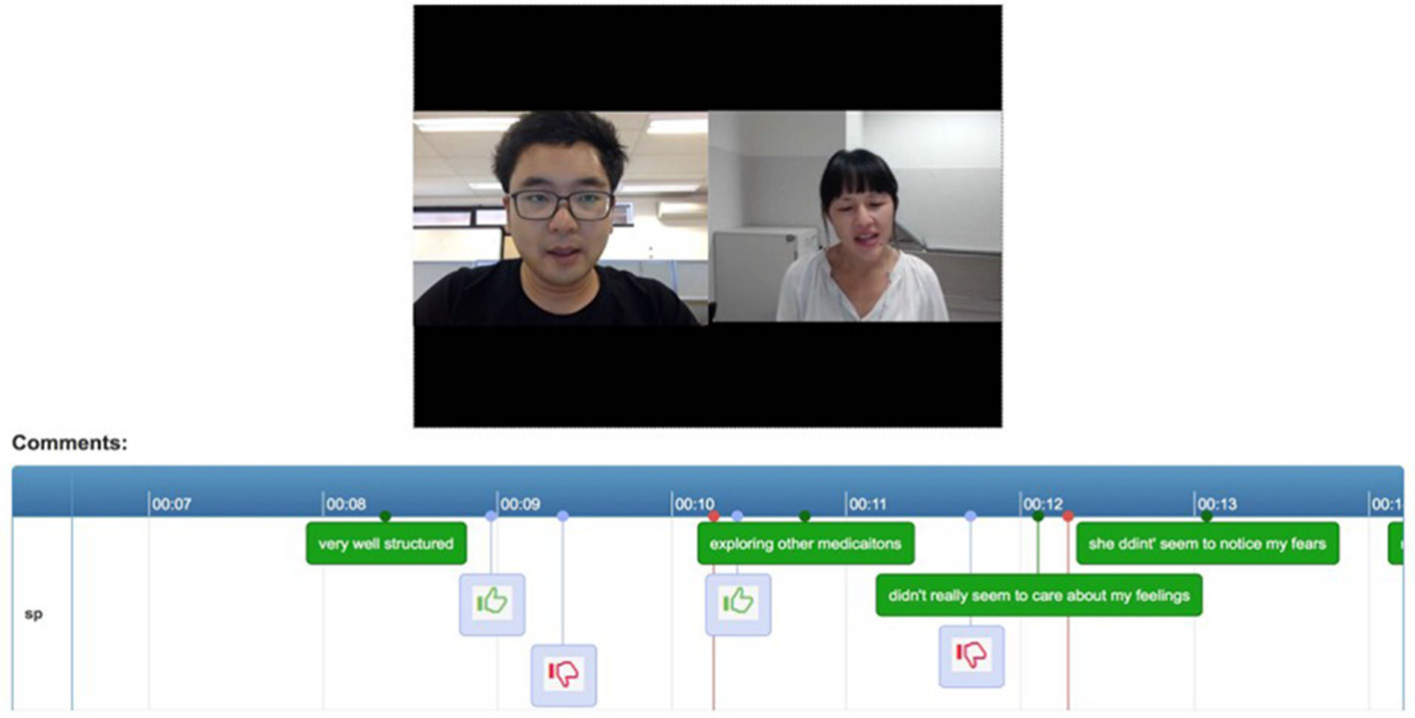
Comment feedback for students.

The tele-consultation component enables students and SPs to have tele-consultations. This component works on most web browsers of a PC or an Android tablet. Once both participants are connected, the system automatically records the consultation. In traditional face-to-face clinical communication skills training, SPs provide overall comments on the performance of students after the consultations. However, using this method, it is difficult to track SPs’ experiences during the consultations. EQClinic attempts to solve this problem by providing two tools for SPs: a ‘thumbs-up’ and a ‘thumbs-down’ tool and a comments box (Fig. 1). The SPs are asked to click the ‘thumbs-up’ button when they feel there is a positive moment in the consultation, and to click the ‘thumbs-down’ button for a negative moment. The SPs are able to input text comments through the comments box during the consultation, indicating positive and negative moments in the consultation. Both forms of feedback are stored with timestamps and can be seen by students when they review the recording (Fig. 2).

The feedback generator of EQClinic detects two categories of non-verbal behaviour using audio processing and computer vision techniques: vocalics behaviour and body movement behaviour. Vocalics behaviour includes volume, pitch, turn-taking patterns, and speaking ratio (a cumulative percentage of the total time each person speaks within a given time frame); body movement behaviour includes nodding; head shaking; facial expressions, such as smiling and frowning; body leaning; head tilting; overall body movements; and face-touch gestures. Each 15-min video recording takes 35 min to be analysed on a personal computer with 3.40 GHz CPU and 16 GB RAM.

**Fig. 3:**
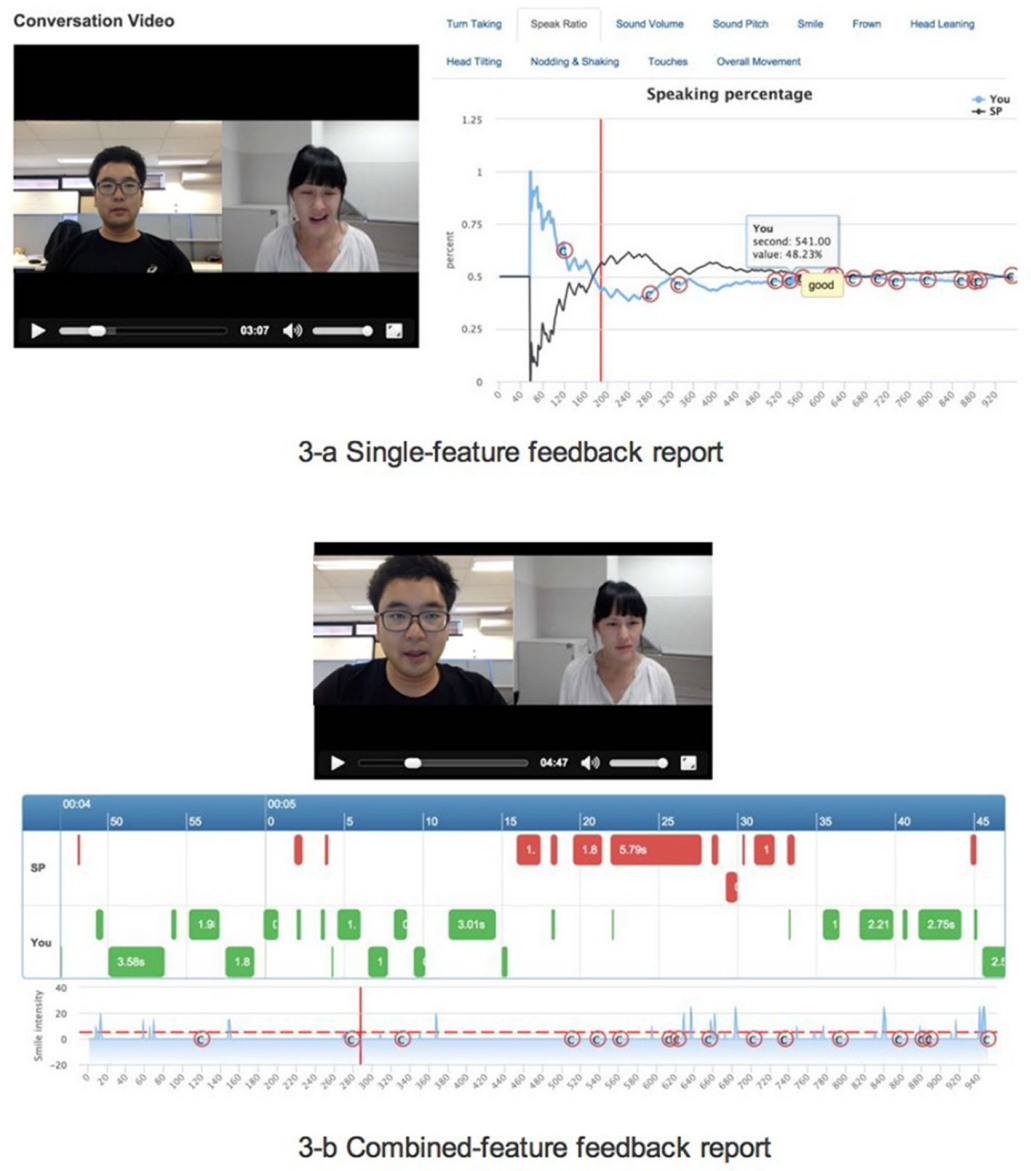
Non-verbal behaviour feedback report.

EQClinic visualises students’ non-verbal behaviour through two types of feedback reports: single-feature (Fig. 3a) and combined-feature reports (Fig. 3b). The single-feature feedback report illustrates each form of non-verbal behaviour separately. Figure 3a is an example of a single-feature feedback report that describes the speaking ratio of the student. From this report, students can easily observe the variations of a particular kind of non-verbal behaviour during a consultation.

The single-feature feedback report helps students to focus on one aspect of non-verbal behaviour, but interactions between different types of non-verbal behaviour are also useful. For example, in the single-feature feedback report, it is difficult to see that a student often shakes his head while smiling. Thus, EQClinic also provides a combined-feature feedback report that displays multiple kinds of non-verbal behaviour on a single page (Fig. 3b).

### Pilot study

We conducted a pilot study using EQClinic in December 2015.

#### Participants

Participants of the pilot study were volunteer SPs and medical students from Years 1 to 4 of UNSW Medicine. The study was approved by the University of Sydney, Human Ethics Research Committee (protocol 2015/151).

#### Instruments

Four questionnaires were used in the study:

*Student–Patient Observed Communication Assessment Form.* An edited version of the Calgary–Cambridge Guide (19) was used by SPs, students, and tutors to assess student performance. It evaluated students with regard to four aspects: providing structure, gathering information, building rapport, and understanding the patient's needs. Each aspect was scored on a four-point scale, in which 1 was the lowest and 4 the highest.

*Confidence Questionnaire.* This aimed to evaluate the students’ confidence in their communication skills on a scale from 1 to 7 through the question ‘How confident do you feel now about your communication skills?’

*Reflection Questionnaire.* This aimed to help students to reflect on the consultations. Students completed it after reviewing the SP's assessment. The free-text questions were:

1. How do you feel the interview went for you at the time?
2. How does this compare with the grade and comments entered by the assessor?
3. How will you continue to develop your communication skills?

*System Usability Questionnaire.* This was a shorter version of the Computer System Usability Questionnaire (20), with questions on a scale from 1 to 7. In addition to the five standard questions, students were asked to identify the most and least useful sections of the non-verbal behaviour feedback.

#### Procedure

At the beginning of this study, three SPs received a 1-h face-to-face training session on EQClinic platform from a researcher involved in this project. During the training session, the researcher demonstrated the procedures of this study using the main components of the platform: booking appointments, having consultations with students, providing real-time comments, and evaluating a student's performance.

Students were expected to be more proficient with technology; therefore, we did not provide them with face-to-face training. Instead, we provided an introductory email with a training video (www.youtube.com/watch?v=cDNZP27fYkM). In that email, we described the details of the study, confirmed consent, and asked students to watch the training video. We also informed them that once they felt comfortable and confident with the system, they could start requesting consultation time slots with an SP on their personal calendar. After their consultation requests had been confirmed, they would have an online conversation with the SP.

Students were asked to conduct two consultations at least 3 days apart (not necessarily with the same SP). The consultations focused on history-taking and could be held anywhere with a good Internet connection and lighting.

During the consultations, the student and the SP first had a 15-min interview through the tele-conference component. After each consultation, the SP assessed the performance of the student using the Assessment Form. The students evaluated their confidence in their communication skills using the Confidence Questionnaire and assessed themselves using the Assessment Form. Then they reviewed the SP's Assessment Form and reflected on the interview using the Reflection Questionnaire.

Since EQClinic took time to analyse the video, the students were asked to return to the system 24 h after the first consultation to review the automated non-verbal behaviour feedback (with SP comments) and fill out the System Usability Questionnaire. A tutor reviewed the consultation videos and assessed the students’ performance using the Assessment Form; these were not seen by the students.

## Results

In total, 13 consultations were completed by eight medical students and three SPs. Five students engaged in two consultations; three students did not undertake a second consultation. We recorded 233 min of consultations. At the start of each consultation was a short introduction between participants. The average length of the consultations was 17.1 min (SD=2.1).

The SP assessments on average produced higher scores than those by the students and tutor (Table 1). However, the gap between the student, tutor, and SP scoring decreased in the second consultations. We did not observe improvements in the overall assessment scores and the SPs’ average assessment scores decreased from 3.25 to 2.9. However, the consultation and feedback helped students identify skills that needed improvement. For example, in the feedback on the first consultation, a SP suggested that the student needed to ask about the patient's main concern at the start of consultations; the student adopted that suggestion in her second consultation, which led to a better conversation structure. The Confidence Questionnaire results showed that the students felt more confident about their communication skills in the second consultations (increased from 3.6 to 4.4) (Table 1).

**Table 1:** Average assessment results

	Length	SP assessment	Tutor assessment	Student self-assessment	Confidence
All 8 students	17.1 min (SD=2.1)	3.00 (SD=0.47)	2.63 (SD=0.38)	2.73 (SD=0.27)	
Five students (who had two consultations)					
First consultation	17.4 min (SD=3.3)	3.25 (SD=0.27)	2.75 (SD=0.39)	2.65 (SD=0.25)	3.6 (SD=1.36)
Second consultation	16.7 min (SD=0.5)	2.9 (SD=0.56)	2.81 (SD=0.11)	2.70 (SD=0.32)	4.4 (SD=0.49)

The students’ feedback in the Reflection Questionnaire indicated that most agreed with the SP's assessment and comments. More than half of the students mentioned that they felt rushed during the consultation and did not have enough time to ask questions or clarify their questions, and they would have liked to have improved their time management. Some students felt confused about organising the structure of the consultation. For example, some students mentioned that they felt unclear about which questions should be asked and in which order, so they spent too much time on unnecessary topics. Two students mentioned that they would have liked to have paid more attention to their non-verbal behaviour, such as nodding and eye contact in their second consultation.

As shown in Table 2, the students’ feedback in the System Usability Questionnaire indicated that they found the feedback on turn taking, smile intensity, frown intensity, and head nodding to be the most useful parts of the non-verbal behaviour feedback, whilst the least useful were feedback related to volume, pitch, and body leaning. Students were positive about the system usability and felt comfortable using it (average=5.9, SD=0.9). They reported that the structure (average=5.4, SD=0.8) and information (average=5.8, SD=1.0) of the system were clear, but it needed to provide clearer instructions when students encountered errors (average=4.4, SD=1.0).

**Table 2:** Response to System Usability Questionnaire

Questions about non-verbal behaviour feedback	
Most helpful non-verbal behaviour feedback sections.	Turn taking, smile intensity, frown intensity, and head nodding
Least helpful non-verbal behaviour feedback sections.	Volume, pitch, and body leaning

Usability questions	Score

I feel comfortable using the system.	5.9 (SD=0.9)
The information provided with this system is clear.	5.4 (SD=1.0)
The structure of this system is clear.	5.9 (SD=0.8)
I like using the interface of this system.	5.3 (SD=0.4)
Whenever I make a mistake using this system, the system made it easy to get back to where I wanted.	4.4 (SD=1.0)

## Discussion

Our pilot study demonstrated that, compared with traditional face-to-face communication skills training programmes, EQClinic was more flexible in organising consultation appointments and facilitated easier set up of the recording environment. The SPs’ assessment clearly pointed out the defects of students’ communication skills and the Reflection Questionnaire promoted student reflection. In addition, the SPs’ real-time comments helped the students to understand their thinking during the consultations.

In this study, we did not observe students’ improvement in the overall assessment scores.

A potential reason for this is that the students did not expect to have the same scenario in their second consultation and were distracted trying to recall the feedback from the first consultation. However, the decreased gaps between the student, tutor, and SP scores in the second consultations indicated that the students had developed a better self-assessment capability. In addition, more students mentioning non-verbal behaviours in the Reflection Questionnaire of their second consultation indicated that EQClinic's non-verbal behaviour feedback helped these students to be more aware of their non-verbal behaviour. However, it is not easy to improve non-verbal communication skills in a short period of time. A previous study provided two groups of medical students with feedback on their Objective Structured Clinical Examination (OSCE) performance from facilitators (9). One group was provided with extra non-verbal behaviour feedback based on a trained non-verbal behaviour model (21). The students who received extra non-verbal behaviour feedback did not improve in their OSCE clinical consultation scores; however, according to student's feedback, those students paid more attention to their non-verbal communication.

As with this previous study, the students in our pilot study did not improve in their overall clinical consultation scores after reviewing automated non-verbal behaviour feedback provided by EQClinic. However, more students mentioned that they would like to improve their non-verbal behaviour in their second consultations, which indicated that EQClinic had enhanced their awareness of the importance of non-verbal communication. Compared with manual forms of annotation of non-verbal behaviours, EQClinic reduced the work needed by students to identify those behaviours.

A limitation of EQClinic's non-verbal behaviour feedback was that it could only identify non-verbal behaviour, rather than evaluate it. According to students’ free-text feedback, they would have liked to receive individualised suggestions about which specific types of non-verbal behaviour they needed to pay attention to and ways in which they could improve them. However, due to the limited number of tele-consultations recorded in this pilot study, it was hard to generate a standard model of non-verbal behaviour and provide valuable non-verbal behaviour evaluation based on that model. With increasing numbers of recorded tele-consultations, we think it is possible to train a model of non-verbal behaviour during tele-consultations.

In the open-ended feedback questionnaire, students pointed to other issues with communication skills training via tele-conferencing. Some found it difficult to maintain eye contact with patients, because they could not physically look into their eyes. Students were also confused about where they should look during the consultation: the middle of the screen or the camera.

This pilot study focused on testing the technology and user acceptance, and collected some initial findings related to student learning. Although our study demonstrated that the system was stable, our next step will need to evaluate the stability and capability of the system with a larger number of users. User acceptance depends on the usability of the system (16), and the results of the System Usability Questionnaire showed that EQClinic achieved positive user acceptance from the students.

The initial findings did not provide enough evidence of the learning gains that can be attributed to using EQClinic. Our next study with a larger cohort will investigate whether using EQClinic improves learning outcomes. If medical students using EQClinic improve their scores in tele-conferencing, we will need to investigate if this learning also improves face-to-face consultations and if there is a particular feedback component that contributes the most benefits. In addition, training a standard model of non-verbal behaviour performance in tele-consultations and providing users with more valuable evaluation and suggestions for improvement are part of our future work.

Other limitations should be considered in this study. The main limitation was that we did not provide a calibration procedure during the SP training session to ensure inter-rater agreement of the assessments. This limitation might have caused inconsistency between the SPs’ assessments of the students and may have contributed to the lack of improvement between the first and second tele-consultations. In addition, the students in this study were from different years (Years 1–4) of medical school and had different levels of knowledge about communication skills, which might have affected their assessment results and ability to reflect on their performance.

In conclusion, EQClinic provides an innovative solution for providing medical students with a means to practise and enhance their communication skills, and eases the work of a medical school organising a training programme. However, its potential influence is not limited to medical education. With the increasing use of telehealth, EQClinic could be incorporated into postgraduate medical training and nurse education programmes to help health professionals to reflect on and develop their communication skills.

## References

[1] Makoul G, Schofield T (1999). Communication teaching and assessment in medical education: an international consensus statement. Patient Educ Couns.

[2] Stewart MA (1995). Effective physician-patient communication and health outcomes: a review. CMAJ.

[3] Mann KV (2011). Theoretical perspectives in medical education: past experience and future possibilities. Med Educ.

[4] Howley LD, Martindale J (2004). The efficacy of standardized patient feedback in clinical teaching. A mixed methods analysis. Med Educ Online.

[5] Novack DH, Cohen D, Peitzman SJ, Beadenkopf S, Gracely E, Morris J (2002). A pilot test of WebOSCE: a system for assessing trainees’ clinical skills via teleconference. Med Teach.

[6] Daetwyler CJ, Cohen DG, Gracely E, Novack DH (2010). eLearning to enhance physician patient communication: a pilot test of ‘doc. com’ and ‘WebEncounter’ in teaching bad news delivery. Med Teach.

[7] Ekman P, Friesen WV, O'Sullivan M, Scherer K (1980). Relative importance of face, body, and speech in judgments of personality and affect. J Pers Soc Psychol.

[8] O'Sullivan M, Ekman P, Friesen W, Scherer KR (1985). What you say and how you say it: the contribution of speech content and voice quality to judgments of others. J Pers Soc Psychol.

[9] Ishikawa H, Hashimoto H, Kinoshita M, Yano E (2010). Can nonverbal communication skills be taught?. Med Teach.

[10] Hartzler A, Patel R, Czerwinski M, Pratt W, Roseway A, Chandrasekaran N (2014). Real-time feedback on nonverbal clinical communication. Methods Inf Med.

[11] Hoque ME, Picard RW (2014). Rich nonverbal sensing technology for automated social skills training. Computer.

[12] Edirippulige S, Armfield N (2016). Education and training to support the use of clinical telehealth: a review of the literature. J Telemed Telecare.

[13] Rienits H, Teuss G, Bonney A (2016). Teaching telehealth consultation skills. Clin Teach.

[14] Liu C, Scott KM, Calvo RA Towards a reflective online clinic (ROC): a tool to support interviewing skills. CHI'15 Workshop on Developing Skills for Wellbeing, Seoul, Korea, April 2015.

[15] Miller EA (2003). The technical and interpersonal aspects of telemedicine: effects on doctor–patient communication. J Telemed Telecare.

[16] Broens TH, Huis in't Veld RM, Vollenbroek-Hutten MMR, Hermens HJ, Van Halteren AT, Nieuwenhuis LJ (2007). Determinants of successful telemedicine implementations: a literature study. J Telemed Telecare.

[17] DeChant HK, Tohme WG, Mun SK, Hayes WS, Schulman KA (1996). Health systems evaluation of telemedicine: a staged approach. Telemed J.

[18] Kolb DA (1984). Experiential learning: experience as the source of learning and development.

[19] Simmenroth-Nayda A, Heinemann S, Nolte C, Fischer T, Himmel W (2014). Psychometric properties of the Calgary Cambridge guides to assess communication skills of undergraduate medical students. Int J Med Educ.

[20] Lewis JR (1995). IBM computer usability satisfaction questionnaires: psychometric evaluation and instructions for use. Int J Hum Comput Interact.

[21] Ishikawa H, Hashimoto H, Kinoshita M, Fujimori S, Shimizu T, Yano E (2006). Evaluating medical students’ non-verbal communication during the objective structured clinical examination. Med Educ.

